# Co-Infection of PRRSV-1 and PRRSV-2 in a Single Sow: Complete Genome Characterization Reveals a Novel CH-1a-Backbone Recombinant with an NADC30-Derived Nsp2 in China

**DOI:** 10.3390/v18091044

**Published:** 2026-09-20

**Authors:** Jiakai Li, Shuo Li, Jie Han, Rui Zhou, Zili Li

**Affiliations:** 1 State Key Laboratory of Agricultural Microbiology, Key Laboratory of Preventive Veterinary Medicine in Hubei Province, College of Veterinary Medicine, Huazhong Agricultural University, Wuhan 430070, China; 2 Key Laboratory of Chemical Veterinary Drug Innovation in Central China, Ministry of Agriculture and Rural Affairs, Wuhan 430060, China

**Keywords:** PRRSV, co-infection, recombination, genome characterization, CH-1a, NADC30, Nsp2

## Abstract

Porcine reproductive and respiratory syndrome virus (PRRSV) has the characteristics of a fast evolution rate and high genetic heterogeneity. PRRSV-1 and PRRSV-2 are co-circulating, and the threat they pose to the health of pigs worldwide continues to rise. In this study, two PRRSV strains with significantly different genetic backgrounds, namely SHEU-2022 (PRRSV-1, full length 15,050 nt) and SHCH-2023 (PRRSV-2, full length 15,019 nt), were simultaneously isolated from a single serum sample of a co-infected sow in Shanghai, China, and their whole-genome characteristics were analyzed. SHEU-2022 clustered with indigenous PRRSV-1 isolates in China, harbored a unique 15-nt deletion in the ORF3-ORF4 overlapping region, carried an additional N-glycosylation site on GP5, and replicated only in porcine alveolar macrophages (PAMs) under the conditions used. SHCH-2023 is a novel recombinant strain with a CH-1a-like (lineage 8) genome backbone and two NADC30-derived fragments—one in Nsp2 (sublineage 1.8) carrying the characteristic 131-amino acid deletion and one in the GP2/E region; it replicated in both PAMs and Marc-145 cells, though this may partly reflect the enrichment procedure. No PRRSV-1/PRRSV-2 recombination was detected between the two co-infected strains.

## 1. Introduction

Porcine reproductive and respiratory syndrome (PRRS) is a highly contagious viral disease which has caused serious economic losses to the global pig industry [1]. Its pathogen is porcine reproductive and respiratory syndrome virus (PRRSV), which can cause acute respiratory disease in growing pigs and severe reproductive disorders in sows, with typical manifestations of late abortion, stillbirth, and weak piglet delivery [2,3]. Initially identified in North America and Europe during the late 1980s, the virus is currently classified by the International Committee on Taxonomy of Viruses (ICTV) into two distinct species within the genus Betaarterivirus: Betaarterivirus suid 1 (commonly referred to as PRRSV-1 or the European type) and Betaarterivirus suid 2 (PRRSV-2 or the North American type) [4,5], which share only 50–60% nucleotide sequence identity at the whole-genome level [5,6,7].

Despite three decades of in-depth research, achieving effective prevention and control of PRRS remains a huge challenge. The main prevention and control obstacles include the extremely high mutation rate, extensive genetic heterogeneity, and strong host immune response escape ability of the virus [8]. Since 2006, the emergence of highly pathogenic PRRSV-2 strains in China has further aggravated the clinical impact of the disease and also updated the academic community’s understanding of virus evolution and transmission dynamics [9]. The overlapping areas of the geographical distribution of PRRSV-1 and PRRSV-2 are increasing, and multiple dual infection cases have been found in the field, including recent cases in China and Japan [10,11], making the prevention and control situation more complicated. Notably, both genotypes have been co-isolated from a single farm and characterized at the whole-genome level [12]. Such co-infection will expand the diversity of viruses, provide a suitable environment for virus recombination, and then generate new variant strains with changed virulence or transmission capacity [13,14]. Beyond co-infection between the two species, recombination also occurs frequently among PRRSV-2 lineages: NADC30-like (sublineage 1.8) strains in particular readily recombine with other lineages, generating a wide array of recombinant variants in China [12].

PRRSV evades host immunity through multiple mechanisms [15,16,17], notably by inhibiting the type I interferon signaling pathway (the core pathway of innate antiviral defense) and by delaying the induction of neutralizing antibodies. These mechanisms promote long-term viremia, increase susceptibility to secondary pathogens, and limit the efficacy of current vaccines. In the field, the protection provided by attenuated live vaccines (MLVs) and inactivated vaccines is often unstable: they typically confer strong homologous protection, but only weak cross-protection against heterologous strains [18]. Continuous antigenic drift further narrows this protection spectrum, reducing cross-reactivity even among strains of the same genotype [19,20].

PRRS is endemic across China, and PRRSV-1/PRRSV-2 co-circulation and co-infection are increasingly reported [10,11]. However, whole-genome data for naturally co-infecting PRRSV-1 and PRRSV-2 isolates from the Yangtze River Delta remain scarce, limiting our understanding of their evolutionary relationships and recombination potential in this high-density pig-producing region. To address this gap, we characterized the complete genomes of SHEU-2022 and SHCH-2023, which were co-isolated from a single sow in Shanghai, and analyzed their phylogenetic relationships and recombination events, revealing SHCH-2023 as a recombinant with a CH-1a-like (lineage 8) backbone carrying NADC30-derived fragments. Co-infection of both genotypes in a single sow also highlights a broader point: in high-density pig-producing areas, control programs can easily fall behind unless supported by regular monitoring and timely data sharing.

## 2. Materials and Methods

### 2.1. Sample Collection

In December 2022, serum samples were collected from 6 sows with suspected clinical symptoms of PRRS in a commercial farm in Shanghai. According to pig farm records and veterinary evaluation, the diseased animals showed transient fever (rectal temperature > 40.5 °C) and decreased feed intake during the 2~3 day observation period. All samples were sent to the internal diagnostic laboratory of the pig farm for preliminary screening. The quantitative reverse transcription PCR (qPCR) test results showed that one sample was positive for both genotypes at the same time: PRRSV-1 (Ct = 19.68) and PRRSV-2 (Ct = 28.77), strongly suggesting the existence of natural co-infection. The remaining five serum samples were all positive for PRRSV-2 only, indicating that PRRSV-2 was the predominant circulating genotype in this herd. Then, this double-positive sample was selected for virus isolation and in-depth genomic characteristic analysis.

Ethics Statement: This study used residual diagnostic serum samples collected for routine disease surveillance. The protocol was reviewed and approved by the Scientific Ethics Committee of Huazhong Agricultural University (Approval No. HZAUSW-2019-027, approved on 10 September 2019).

### 2.2. Primer Design and Synthesis

To achieve complete genome amplification, we designed genotype-specific primer sets based on representative reference strains included in NCBI GenBank. For PRRSV-2, the full-length sequences of CH-1a (AY032626), JXA1 (EF112445) and NADC30 (JN654459) were aligned to determine the conserved regions. Oligo 7.0 software was used to design a specific primer pair (Nsp2-F/R) targeting the highly variable Nsp2 region, and 12 pairs of overlapping primers were additionally designed to cover the full-length genome (~15 kb) of PRRSV-2, as detailed in Table 1.

For the detection and sequencing of PRRSV-1, primers were designed based on the sequence alignment results of Lelystad virus (NC_043487) and BJEU06-1 (GU047344) strains. The diagnostic primer set includes a specific primer pair (ORF7-F/R) targeting the highly conserved ORF7, as well as 12 pairs of overlapping primers covering the whole genome. All primers were synthesized by Sangon Biotech (Shanghai, China) and verified for specificity with control plasmids before use.

### 2.3. RNA Extraction and RT-PCR Amplification

**Figure 1:**
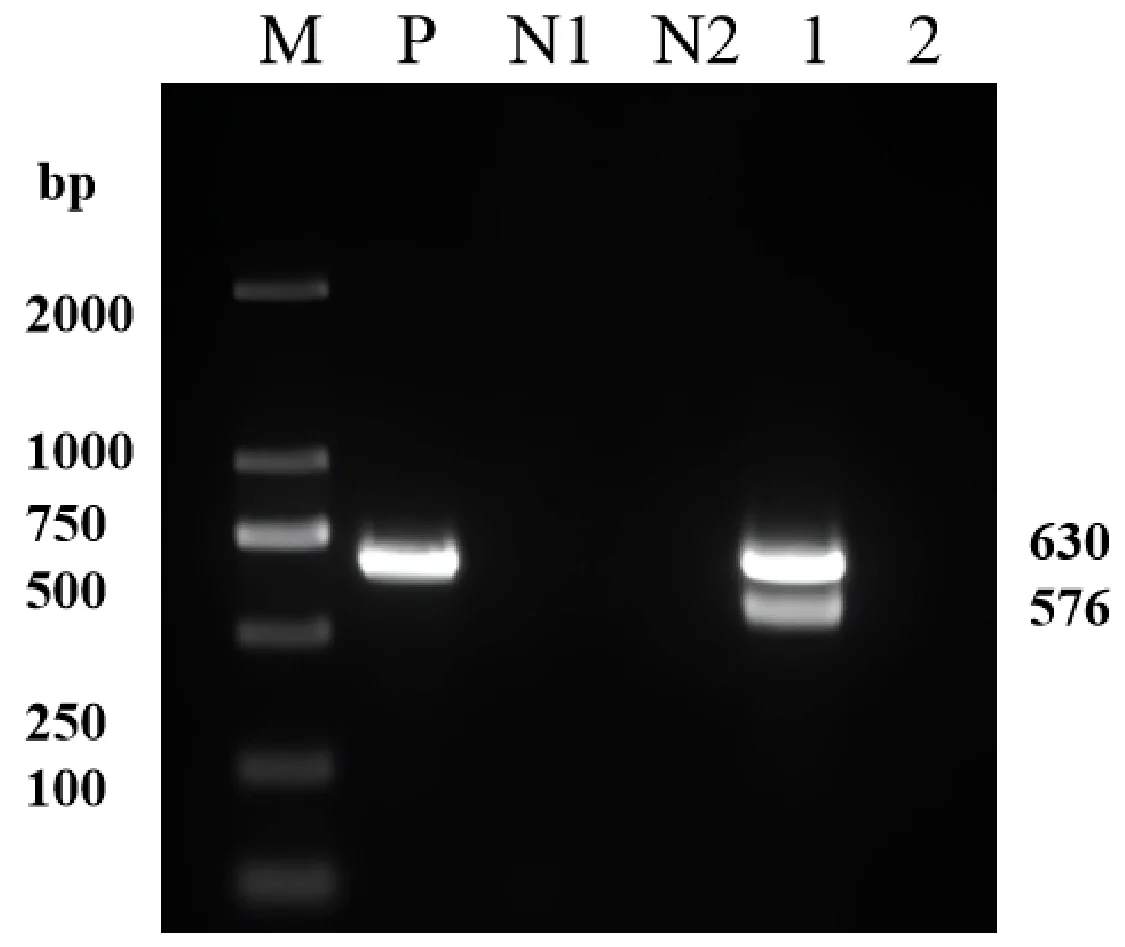
RT-PCR amplification results of PRRSV-2 Nsp2 and PRRSV-1 ORF7 used for differential diagnosis of blood samples in the Shanghai area. M: DL2000 Marker; P: (Nsp2 of NADC30-like in PRRSV-2, 630 bp); N1: PRRSV-2 Nsp2 negative control; N2: PRRSV-1 ORF7 negative control; 1: Serum to be tested; 2: Negative serum.

In strict accordance with the kit instructions, viral RNA was extracted from 140 µL of double-positive serum using the TIANamp Virus RNA Kit (DP315; Tiangen Biotech, Beijing, China), and reverse-transcribed into cDNA with random hexamers using the PrimeScript^TM^ RT Reagent Kit (TaKaRa Bio Inc., Dalian, China). Conventional RT-PCR detection was performed independently for PRRSV-1 and PRRSV-2 using the ORF7-F/R and Nsp2-F/R primer pairs, respectively, with 2× PrimeSTAR^®^ Max Premix (TaKaRa Bio Inc., Dalian, China). The 25 µL reaction contained 12.5 µL of 2× PrimeSTAR Max Premix, 1 µL each of forward and reverse primers (10 µM), 2 µL of cDNA template, and nuclease-free water to 25 µL. The thermal cycling conditions were: initial denaturation at 98 °C for 5 min; followed by 35 cycles, each cycle consisting of denaturation at 98 °C for 10 s, annealing at 55 °C for 15 s, and extension at 72 °C for 30 s; and a final extension at 72 °C for 5 min. The amplification products were separated by 1% agarose gel electrophoresis, stained with GelRed, and visualized under ultraviolet light (Figure 1). Target bands of the expected size (ORF7 ≈ 570 bp; Nsp2 ≈ 630 bp) were excised, purified, and sequenced by bidirectional Sanger sequencing, which simultaneously detected PRRSV-1 and PRRSV-2 in the serum and confirmed the dual infection.

For whole-genome sequencing, total RNA was extracted from the P2 isolates using the TIANamp Virus RNA Kit (DP315; Tiangen Biotech, Beijing, China), and reverse-transcribed into cDNA. Each genome was amplified as 12 overlapping fragments (Table 1) using 2× PrimeSTAR Max Premix. Primers were designed against conserved regions of circulating PRRSV sequences and therefore required no further optimization; primer pairs yielding weak or nonspecific amplification were optimized by gradient PCR (annealing temperature, 55–60 °C), and fragments that failed to amplify were re-amplified after adjusting the annealing temperature, extension time, or cycle number. Amplicons were purified and subjected to bidirectional Sanger sequencing on an Applied Biosystems 3730xl capillary sequencer (Applied Biosystems, Foster City, CA, USA); no next-generation sequencing was used. Reads were assembled with DNASTAR 7.1; each position was read at least twice.

### 2.4. Bioinformatics and Phylogenetic Analyses

Nucleotide sequences were aligned using MAFFT, and the best-fit nucleotide substitution model (GTR+G+I) was selected in MEGA 11 [21]. Phylogenetic trees were constructed by the maximum-likelihood (ML) method in MEGA 11 with 1000 bootstrap replicates. To resolve within-species relationships, separate analyses were performed for PRRSV-1 and PRRSV-2. For both genotypes, trees were constructed based on the complete genome and ORF5; for PRRSV-2, an additional Nsp2-based tree was constructed to resolve the recombinant ancestry. PRRSV-1 strains were classified into subtypes 1–4 according to [22], and PRRSV-2 strains were classified into lineages and sublineages (e.g., lineage 1.8, lineage 8) according to [23]. Reference strains were selected to represent the major PRRSV-1 subtypes and PRRSV-2 lineages, including European and Chinese isolates, together with the recently reported Chinese co-infection strains XJ/PRRSV1/2025 (PX789659) and XJ/PRRSV2/2025 (PX789660), the Anhui co-infection strains AHB1 (PRRSV-1) and Chah2022 (PRRSV-2), and the Japanese co-infection ORF5 sequences 020-P4-EU (LC877719) and 020-P4-NA (LC877720).

Potential recombination events were identified using RDP4 [24] with seven detection algorithms (RDP, GENECONV, BootScan, MaxChi, Chimaera, SiScan, and 3Seq); events supported by at least five algorithms with a Bonferroni-corrected *p* < 0.01 were considered significant. Candidate parental strains were selected based on the highest nucleotide sequence identity to the query together with representative lineage/subtype strains. Recombination breakpoints and genomic similarities were further verified using SimPlot 3.5.1 [25] with a 200-nt sliding window, a 20-nt step size, and the Kimura 2-parameter model. BootScan analyses (100 bootstrap replicates, neighbor-joining criterion) were used to confirm recombination breakpoints, and a similarity plot was used to assess the SHEU-2022 genome. Amino acid variations, potential N-glycosylation sites of GP5, and antigenic epitopes of the N protein were analyzed using the MegAlign and Protean modules of DNASTAR 7.1.

### 2.5. Virus Isolation and Amplification

#### 2.5.1. Preliminary Isolation and Primary Amplification (P1)

The diagnostic serum sample was filtered and sterilized through a 0.22 µm filter membrane. Primary PAMs were inoculated into 6-well culture plates at a density of 1 × 10^6^ cells/mL and incubated until the confluence reached about 80%. The cell monolayer was washed twice with sterile phosphate-buffered saline (PBS, pH 7.4), and then inoculated with 1 mL of filtered serum. After adsorption at 37 °C under 5% CO_2_ for 2 h, the inoculum was aspirated. Then Dulbecco’s Modified Eagle Medium (DMEM) supplemented with 2% fetal bovine serum (FBS) was added to the cells. The cytopathic effect (CPE) of the culture was observed daily. When widespread CPE (involving 70~80% of the cell monolayer) appeared, the culture supernatant was collected. Centrifugation was performed at 500× *g* at 4 °C for 10 min to precipitate cell debris, and the obtained clarified supernatant was P1, which was aliquoted and stored at −80 °C.

#### 2.5.2. Enrichment of PRRSV-2 on Marc-145 Cells

To specifically enrich the PRRSV-2 component, the P1 virus solution was inoculated onto Marc-145 cells, which are highly susceptible to PRRSV-2 but poorly susceptible to PRRSV-1. The Marc-145 monolayer (80% confluence) was inoculated with a 1:10 dilution of the P1 virus solution; after adsorption, the medium was replaced with maintenance medium. When obvious CPE appeared, the supernatant was collected and clarified, yielding the PRRSV-2-enriched Marc-145 passage.

#### 2.5.3. Limiting-Dilution Purification (P2)

Final purification of PRRSV-2: The Marc-145-enriched virus solution was serially diluted 10-fold and inoculated into Marc-145 monolayers (100% confluence) in 96-well plates. After 5–7 days, wells showing a single discrete CPE lesion were selected, and the isolates were amplified once in Marc-145 cells to obtain the purified PRRSV-2 isolate (P2).

Isolation of PRRSV-1: In parallel, the P1 virus solution was serially diluted and inoculated into PAM monolayers in 96-well plates. Wells showing a single CPE lesion were selected, and the isolates were inoculated into fresh PAM and Marc-145 cells for a cross-infection test. The isolate population that induced CPE in PAMs but failed to replicate in Marc-145 cells was identified as the candidate PRRSV-1 isolate and amplified in PAMs to obtain the purified PRRSV-1 isolate (P2).

This difference in cell tropism may be partly derived from the artificial selection of the screening method, rather than the inherent characteristics of the virus itself.

#### 2.5.4. Virus Identification and Stock Preparation

Viral RNA was extracted from positive isolates, reverse-transcribed into cDNA, and genotype-specific PCR was carried out using the above primer sets. If it can only be amplified by PRRSV-1 or PRRSV-2 primers, the genotype of the isolate can be clearly determined. The amplification products were purified and sequenced, and the final identity was determined by BLASTn (BLAST+ version 2.15.0) alignment in the GenBank database. To ensure that the purity of the isolate absolutely meets the standard, PCR-positive samples need to undergo the final round of end-point dilution purification in PAM cells. Through this rigorous process, 2 strains of virus solutions with extremely high purification degree were finally obtained: SHEU-2022 (PRRSV-1) and SHCH-2023 (PRRSV-2). An aliquot of the P2 isolate of each virus was used for whole-genome sequencing. For infectivity determination, the two isolates were further passaged once to obtain the third-generation (P3) stocks, which were serially diluted and titrated on fresh PAM monolayers; the 50% tissue culture infectious dose (TCID_50_) was calculated by the Reed–Muench method.

## 3. Results

### 3.1. Whole-Genome Sequencing and Analysis of the Two Strains

DNASTAR 7.1 software was used to perform seamless splicing of the obtained genome fragments to obtain the complete genomes of the two isolates, and their integrity was verified by BLAST. Because Sanger sequencing was used, each genome position is covered by a bidirectional read from overlapping amplicons rather than by next-generation sequencing coverage depth. The genome length of SHEU-2022 is 15,050 nt, which has been submitted to GenBank with the accession number PQ306310.1. The genome length of SHCH-2023 is 15,019 nt, with the GenBank accession number PQ316100.1. The virus titer was determined by the Reed–Muench method; the infective titer of SHEU-2022 was 10^5.47^ TCID_50_/100 μL, and the infective titer of SHCH-2023 was 10^4.37^ TCID_50_/100 μL.

The comparative analysis results of genome structural parameters showed that the fragment length of SHEU-2022 is completely consistent with the reported characteristics of PRRSV-1, but there are significant differences from PRRSV-2 strains. In contrast, the structural parameters of SHCH-2023 conform to the typical characteristics of PRRSV-2. As summarized in Table 2, the genome length comparison between the two strains and reference strains provides a solid genomic verification for the preliminary classification results.

### 3.2. Whole Genome Sequence Analysis of SHEU-2022 Strain

#### 3.2.1. 5′UTR Sequence

The 5′ untranslated region (5′UTR) of SHEU-2022 is 221 nt in length, containing a highly conserved CACCC region, which is a key regulatory element for transcription factor binding. The 5′UTR sequence homology between SHEU-2022 and PRRSV-1 strains is 87.3~95.9%, with the highest homology to the domestic isolate LNEU12. In comparison, its homology with PRRSV-2 strains is only about 46%, as detailed in Table 2.

#### 3.2.2. Non-Structural Protein Coding Gene Sequence

The ORF1a of the SHEU-2022 strain is 7155 nt in length, with 78.2~85.6% sequence similarity to PRRSV-1 strains, and lower homology to PRRSV-2 strains, only 47.7~49.2%. ORF1b is 4392 nt in length, with 78.2~85.4% sequence similarity to PRRSV-1 strains, and its similarity to PRRSV-2 strains decreases to 58.4~59.4%.

**Figure 2:**
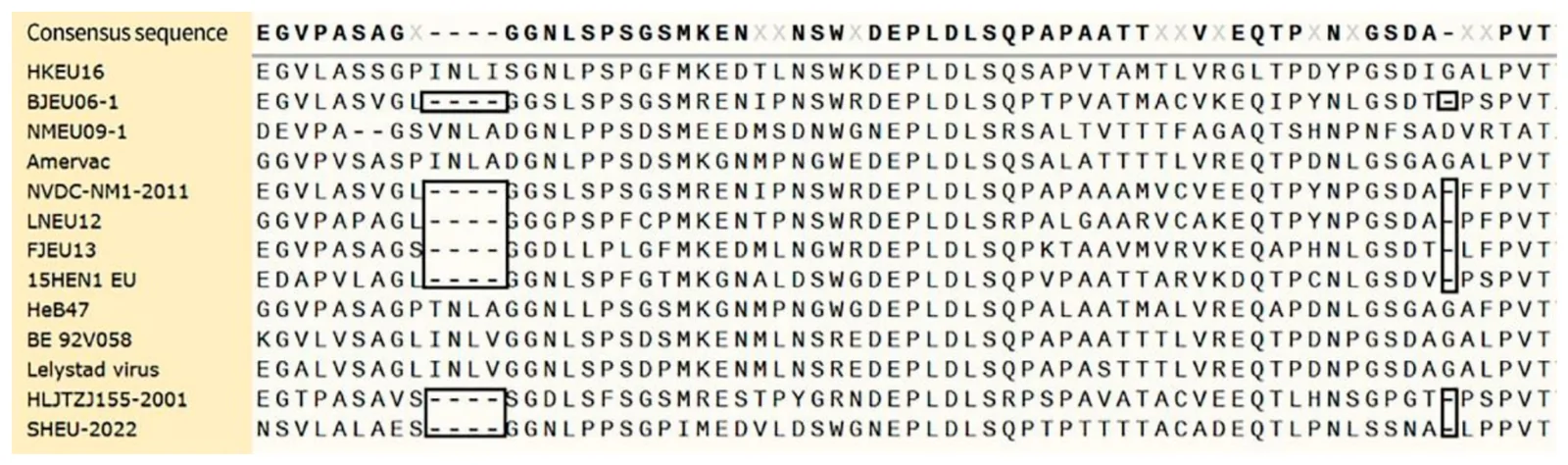
Distribution of amino acid deletions in Nsp2 region of SHEU-2022 strain. Dashes (-) indicate deletions relative to the consensus sequence shown at the top. The alignment illustrates the distribution of amino acid deletions in the Nsp2 region of SHEU-2022 compared with other strains.

In addition to strict nucleotide homology, structural variation of the proteome also has important reference value. Compared with the prototype strain Lelystad virus (LV), the non-structural protein Nsp2 of the SHEU-2022 strain has two independent deletion fragments, with a total deletion of 5 amino acids (Figure 2). Alignment results with previously isolated strains confirm that the Nsp2 domain is the core hotspot region of genetic plasticity of PRRSV-1 strains prevalent in China.

#### 3.2.3. Structural Protein Coding Gene Sequence

**Figure 3:**
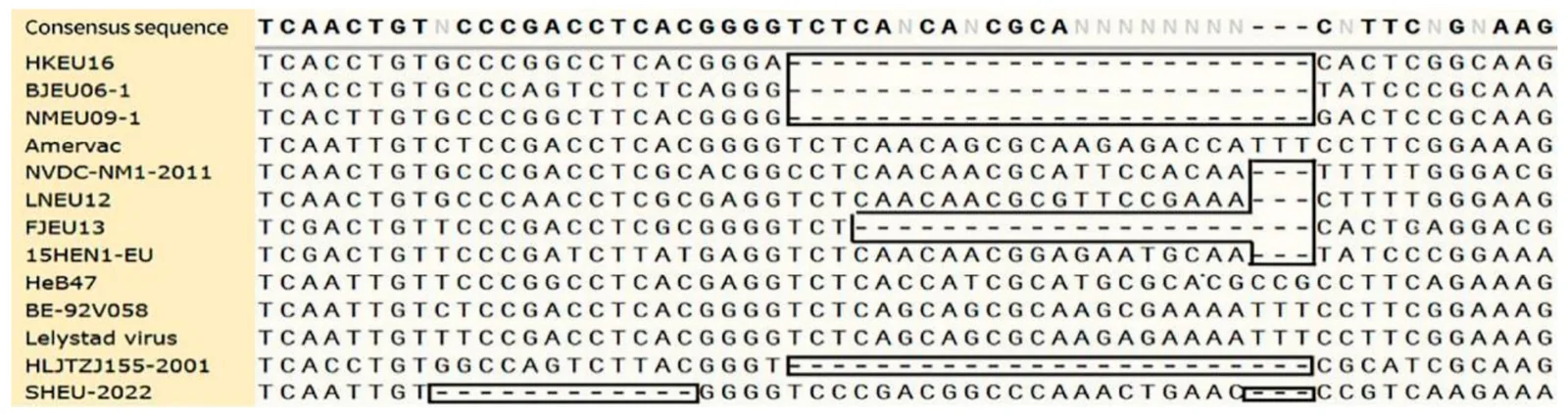
Deletion sites of overlapping gene sequences of ORF3 and ORF4 of SHEU-2022 strain. Dashes (-) indicate deletion sites, illustrating the distribution of deletions within this overlapping gene region.

As shown in Table 3, the structural protein coding gene sequences of the SHEU-2022 strain have high homology with the corresponding sequences of 15 European PRRSV strains. ORF6 and ORF2 are relatively conserved, with nucleotide sequence homologies of 88.1~92.1% and 85.2~92.6%, respectively. The homology of ORF7 ranks next, ranging from 84.4% to 89.2%. On the contrary, the variation degree of ORF3 to ORF5 is significantly higher. In addition, the structural proteins of SHEU-2022 differ greatly from those of PRRSV-2 strains; even for the highly conserved ORF6, the maximum homology is only 70.4%. Critically, genome alignment results show that there is a clearly localized 15 nt deletion in the overlapping region of ORF3 and ORF4 of SHEU-2022 (Figure 3). Further analysis shows that 8 domestic PRRSV-1 isolates have deletions of varying lengths at this site (spanning 3~24 nt), which strongly suggests that this region is a characteristic hypervariable region in the evolution process of PRRSV-1 in China.

#### 3.2.4. Partial Structural Protein Sequence Analysis

**Figure 4:**
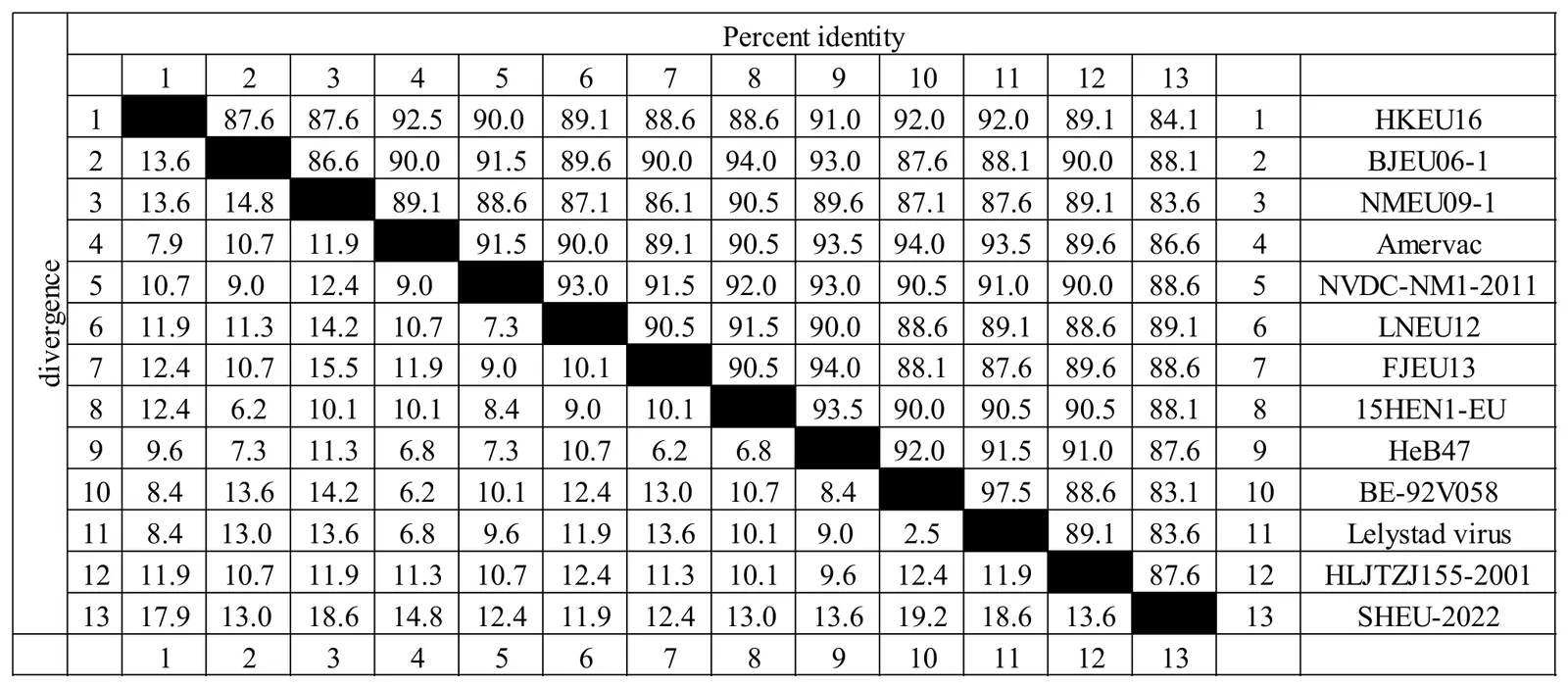
Alignment analysis of ORF5 amino acid sequences of SHEU-2022 and other PRRSV-1 strains. The pairwise comparison was conducted using DNASTAR 7.1 software (DNASTAR Inc., Madison, WI, USA). The matrix presents two types of values, both expressed as percentages (%): the upper-right triangle shows percent identity, and the lower-left triangle shows percent divergence. Higher identity values indicate greater sequence similarity and closer phylogenetic relatedness, whereas higher divergence values reflect greater amino acid differences and genetic heterogeneity between the corresponding strains. Strain numbers (1–13) correspond to the labels listed on the right side of the figure, with number 13 designating the isolate SHEU-2022 from the present study.

Using DNASTAR 7.1 software, the amino acids encoded by ORF5 of the SHEU-2022 strain were aligned with the corresponding sequences of LV, Amervac, and 12 domestic and foreign PRRSV-1 isolates. The results showed that ORF5 of SHEU-2022 encodes 201 amino acids. The alignment results indicate that there are 23 amino acid variations compared with the Lelystad virus strain, with a homology of 83.7%; 27 variations compared with the Amervac strain, with a homology of 86.6%; and the sequence homology with domestic isolates is 83.1~89.1% (Figure 4).

**Figure 5:**
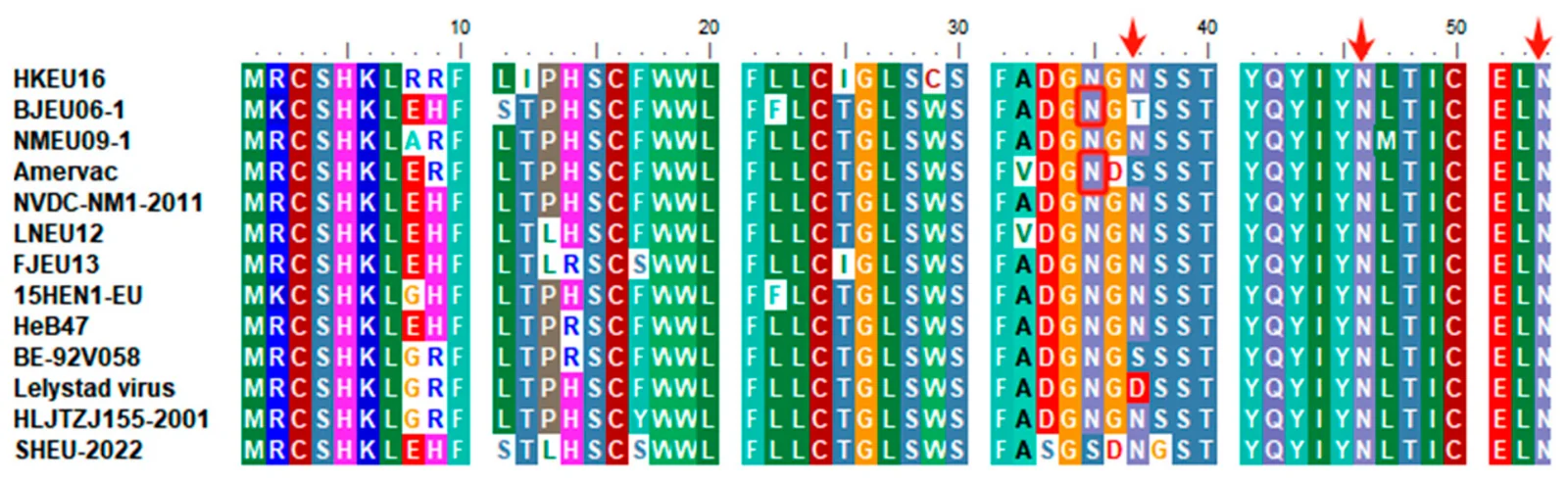
Alignment analysis of the ORF5 amino acid sequences of SHEU-2022 and other PRRSV-1 strains. Red arrows indicate specific amino acid residues, where “N” represents a potential N-glycosylation site.

Analysis using the NetNGlyc online prediction tool found that the GP5 protein encoded by ORF5 of the SHEU-2022 strain has 3 potential N-glycosylation sites, located at amino acids 37~39, 46~48, and 53~55, respectively. In comparison, BJEU06-1, Amervac, BE-92V058, and Lelystad virus strains only have 2 potential N-glycosylation sites. In addition, another 8 domestic PRRSV-1 isolates also have 3 potential N-glycosylation sites simultaneously (Figure 5).

**Figure 6:**
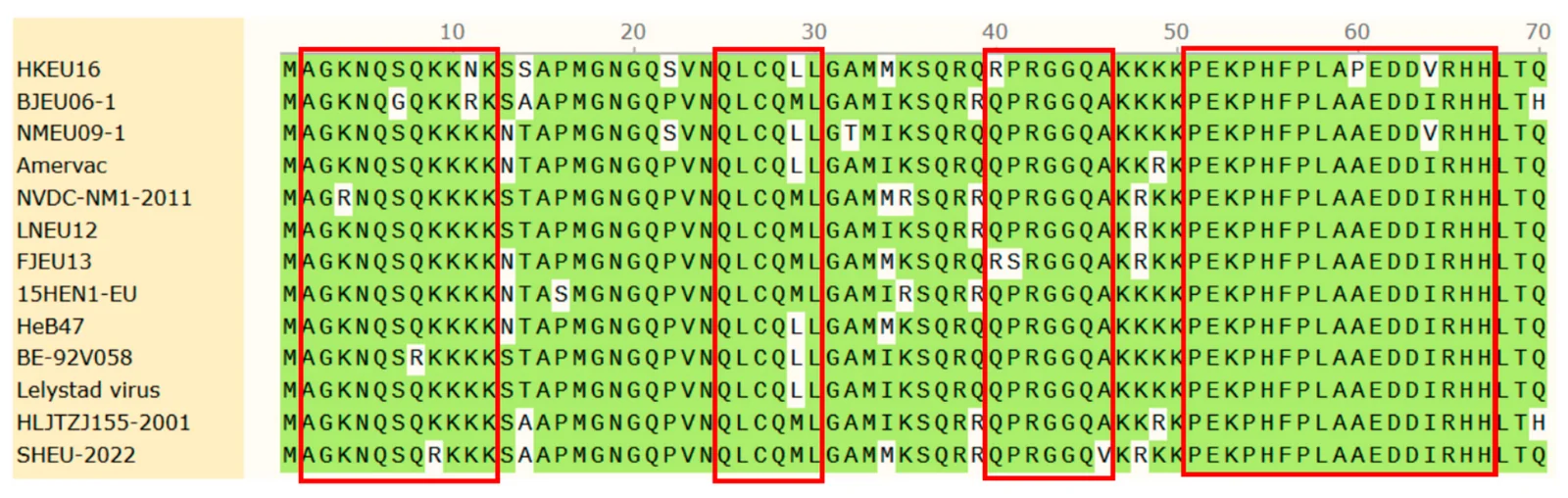
Alignment analysis of N protein antigenic sites of SHEU-2022 and other PRRSV-1 strains. The red boxes highlight the conserved antigenic epitope regions within the N protein. The alignment reveals that SHEU-2022 shares a high degree of sequence conservation in these key antigenic sites compared to the other reference PRRSV-1 strains.

Analysis of the ORF7-encoded amino acid sequence. The ORF7 of the SHEU-2022 strain is 387 bases in full length, translated into the N protein of 128 amino acids, which is recognized as the structural protein with the highest conservation. N protein contains 4 main antigenic determinants: antigenic epitopes A, B, and C are linear epitopes, and antigenic epitope D is a conformational epitope cluster. The sequence of antigenic epitope A of SHEU-2022 is AGKNQSQKKK, antigenic epitope B is QLCQML, and antigenic epitope C is QPRGGQA. Antigenic epitope D covers residues 51~67 and 80~90. Although antigenic epitopes A and C are usually conserved molecular markers of the PRRSV-1 genotype, compared with the consensus sequence, SHEU-2022 has obvious point mutations at the 9th residue of antigenic epitope A and the 46th residue of the full-length sequence corresponding to antigenic epitope C (Figure 6).

#### 3.2.5. Genetic Evolution Analysis of the Full Genome of SHEU-2022

**Figure 7:**
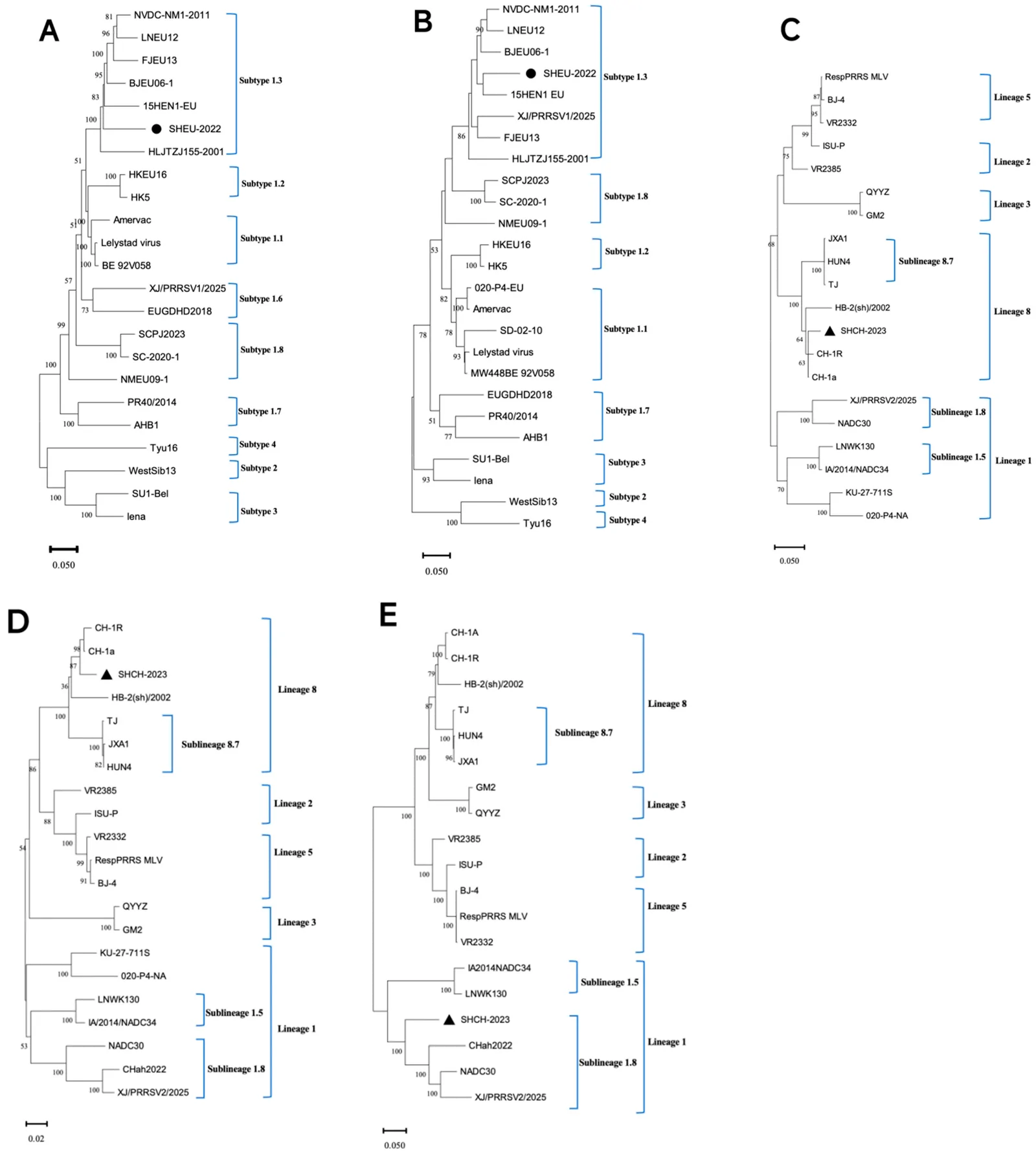
Phylogenetic analysis of the study strains with representative PRRSV reference sequences. Maximum-likelihood (ML) trees were constructed using MEGA 11 with the best-fit nucleotide substitution model (GTR+G+I) and 1000 bootstrap replicates (bootstrap values ≥ 50% are shown at the nodes). (**A**) PRRSV-1 whole genome; (**B**) PRRSV-1 ORF5; (**C**) PRRSV-2 whole genome; (**D**) PRRSV-2 ORF5; (**E**) PRRSV-2 Nsp2. The two strains characterized in this study are marked with black filled symbols: SHEU-2022 (GenBank PQ306310, filled circle) and SHCH-2023 (GenBank PQ316100, filled triangle). PRRSV-1 strains were classified into subtypes 1–4 according to Stadejek et al. [22], and PRRSV-2 strains were classified into lineages and sublineages according to Shi et al. [23]. Scale bars indicate nucleotide substitutions per site.

To clarify the evolutionary status of SHEU-2022, whole-genome and ORF5 phylogenetic trees were constructed using MEGA 11 with representative PRRSV-1 reference sequences. As shown in Figure 7A,B, SHEU-2022 grouped within Subtype 1 (pan-European), in the same Chinese sub-group as BJEU06-1, 15HEN1-EU, FJEU13, HLJTZJ155-2001, LNEU12, and NVDC-NM1-2011. Whole-genome phylogeny placed SHEU-2022 as the sister of 15HEN1-EU with strong support (bootstrap 100), identifying 15HEN1-EU as its closest relative. The ORF5 tree was consistent, grouping the two together, although this node received < 50% bootstrap support owing to the limited signal of the 606-nt ORF5 alignment. This Chinese sub-group forms a sister clade to the European prototype strains (Lelystad virus and Amervac) within Subtype 1, indicating that SHEU-2022 belongs to the Chinese Subtype 1 cluster rather than the Western European prototype strains.

### 3.3. Gene Sequence Analysis of the SHCH-2023 Strain

**Figure 8:**
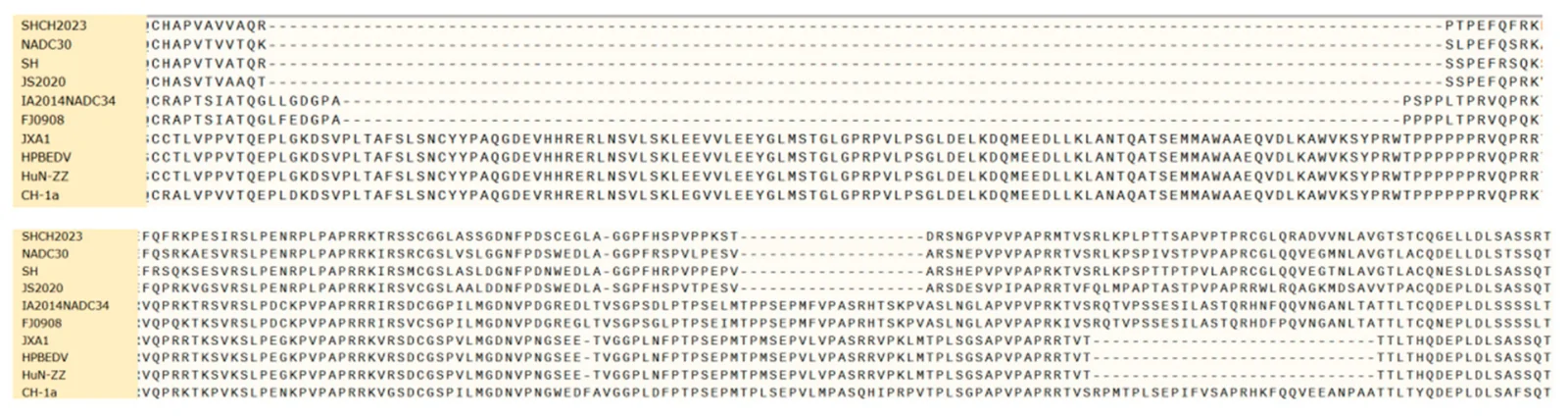
Amino acid deletion pattern in Nsp2 of SHCH-2023 compared with reference PRRSV strains. Multiple sequence alignment reveals the specific amino acid deletion regions in the Nsp2 protein. SHCH-2023 possesses a discontinuous Nsp2 deletion pattern identical to that of the NADC30-like strains, indicating its close genetic relationship with the NADC30-like lineage.

After amplification and translation of the Nsp2 region of SHCH-2023, a significant topological feature was found. Alignment with GenBank reference strains showed that there are 3 discontinuous deletions in the Nsp2 sequence of SHCH-2023, with a total deletion of 131 amino acids, which are 111, 1, and 19 amino acid deletions, respectively. This pattern is consistent with the molecular characteristics of the PRRSV NADC30 strain, as detailed in Figure 8.

**Figure 9:**
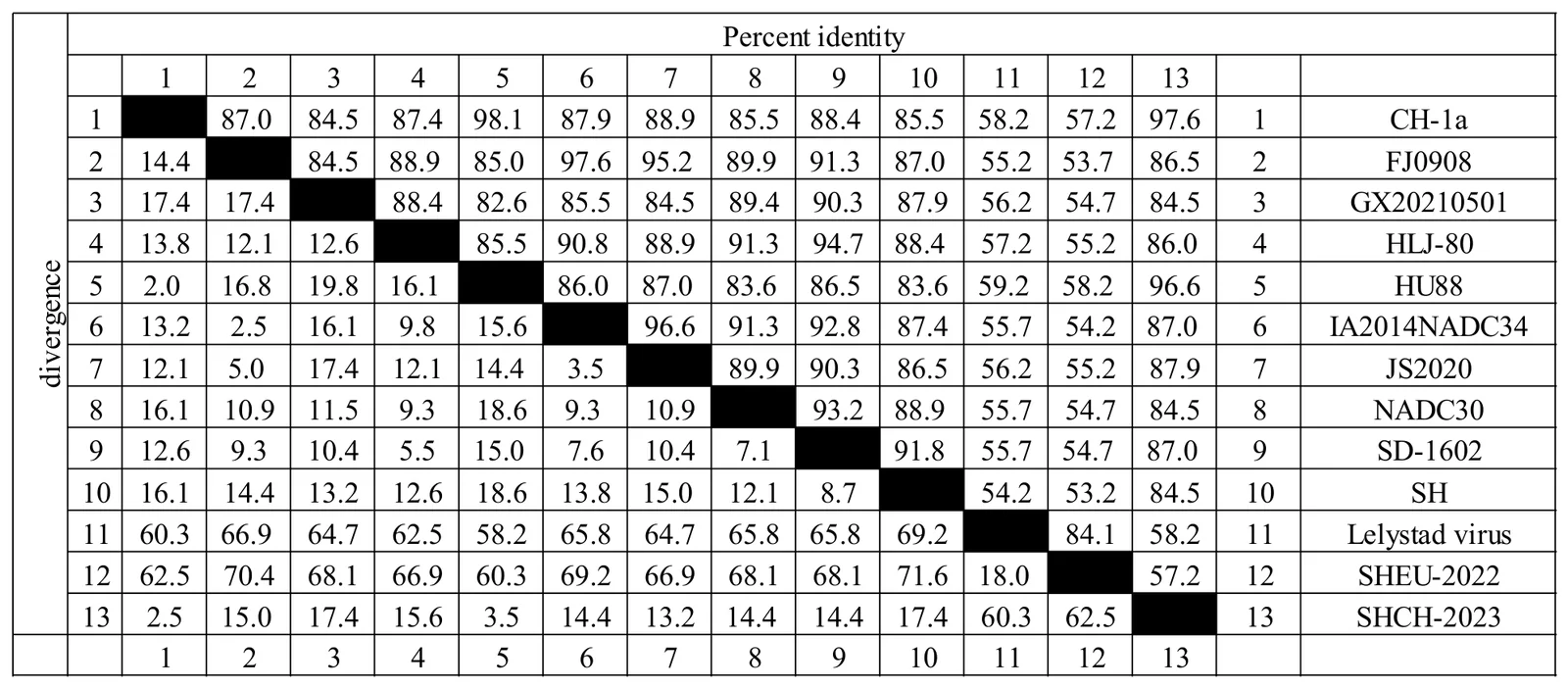
Alignment analysis of ORF5 amino acid sequences of SHCH-2023 and other PRRSV strains. The pairwise comparison was conducted using DNASTAR 7.1 software (DNASTAR Inc., Madison, WI, USA). The matrix presents two types of values, both expressed as percentages (%): the upper-right triangle shows percent identity, and the lower-left triangle shows percent divergence. Higher identity values indicate greater sequence similarity and closer phylogenetic relatedness, whereas higher divergence values reflect greater amino acid differences and genetic heterogeneity between the corresponding strains. Strain numbers (1–13) correspond to the labels listed on the right side of the figure, with number 13 designating the isolate SHCH-2023 from the present study.

On the other hand, DNAstar software was used to analyze the sequence of ORF5 (encoding 201 amino acids) of SHCH-2023, and the results showed that its homology with the classical PRRSV-2 strain CH-1a is as high as 97.6%. Its homology with other PRRSV-2 strains is 84.5~96.6%, and its sequence homology with PRRSV-1 strains is the lowest, about 57.2~58.2% (Figure 9). Based on the ORF5 gene sequence homology alignment results, ORF5 of SHCH-2023 is more closely related to classical PRRSV-2 strains such as CH-1a.

To clarify the evolutionary status of SHCH-2023, whole-genome, ORF5, and Nsp2 phylogenetic trees were constructed using MEGA 11 with representative PRRSV-2 reference sequences obtained from GenBank. As shown in Figure 7C–E, in the whole-genome and ORF5 trees, SHCH-2023 grouped within lineage 8 (CH-1a-like), with CH-1a as its closest relative. In contrast, in the Nsp2 tree, SHCH-2023 grouped within sublineage 1.8 (NADC30-like), with NADC30 as its closest relative. This discordance between the whole-genome/ORF5 and Nsp2 phylogenetic patterns strongly suggests that SHCH-2023 has undergone a gene recombination event.

### 3.4. Recombination Analysis of Two Strains

**Figure 10:**
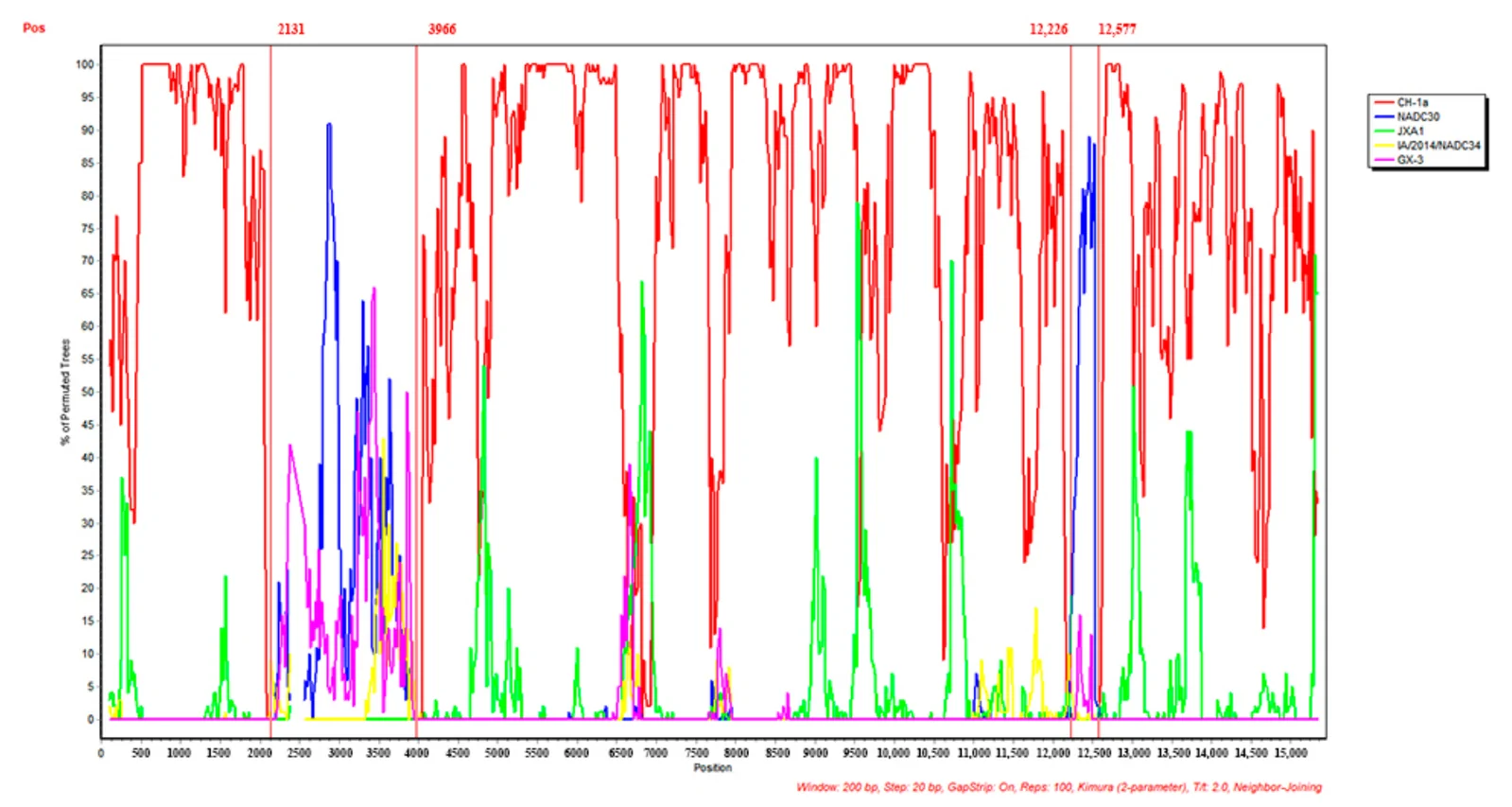
BootScan analysis of the SHCH-2023 genome. The SHCH-2023 sequence was used as the query against five PRRSV-2 reference sequences (CH-1a, NADC30, JXA1, IA/2014/NADC34, and GX-3). The analysis was performed with a 200-nt window, a 20-nt step, the Kimura 2-parameter model, and 100 bootstrap replicates under the neighbor-joining criterion. The y-axis shows the percentage of bootstrap replicates in which SHCH-2023 clustered with each reference sequence. Two NADC30-derived regions are indicated by vertical dashed lines: nt 2131–3966 (Nsp2) and nt 12,226–12,577 (GP2/E), relative to CH-1a. The gap at approximately nt 2400–2540 corresponds to the NADC30 Nsp2 deletion removed by GapStrip.

**Figure 11:**
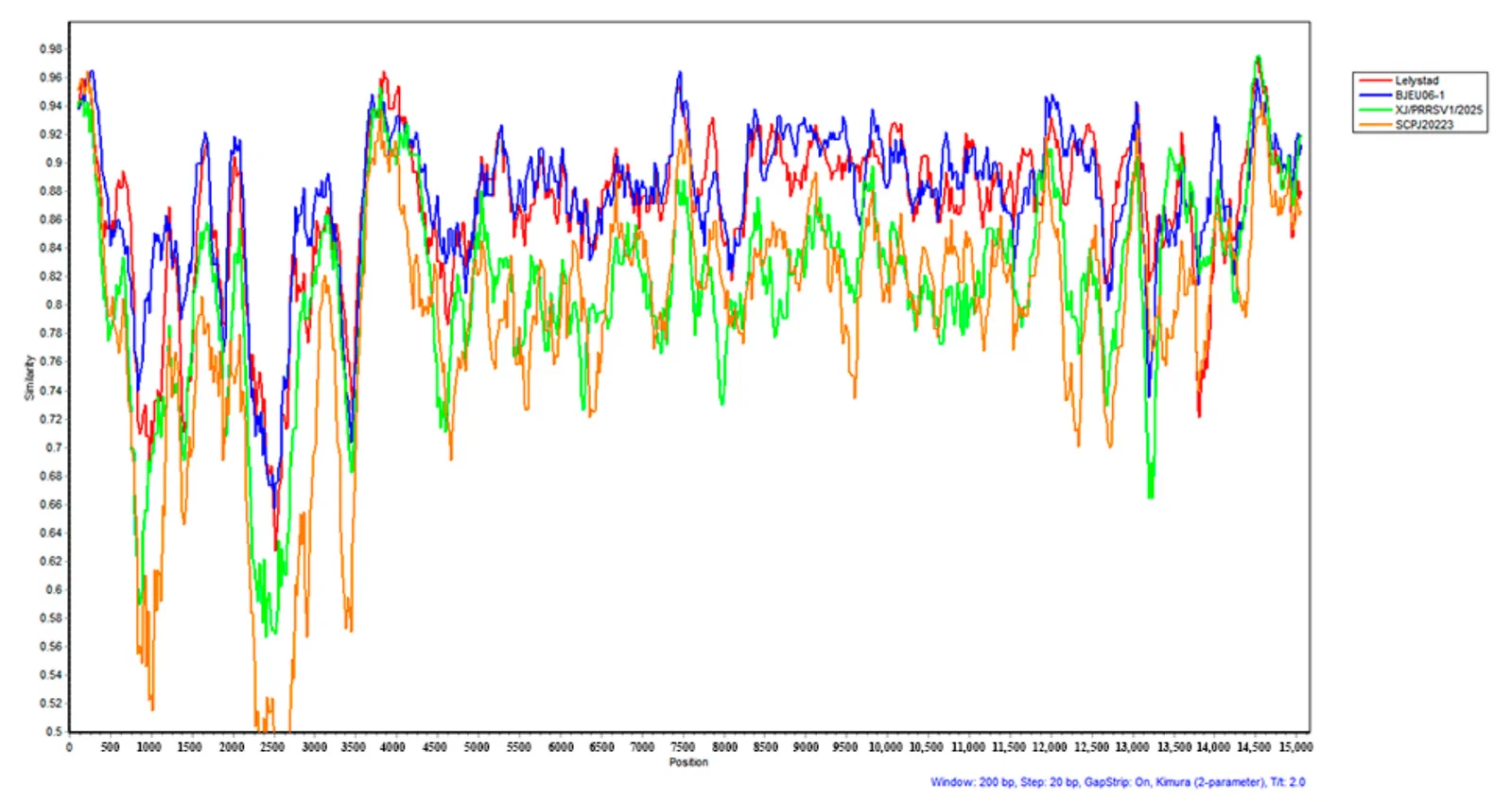
Similarity plot of the SHEU-2022 genome. SHEU-2022 was used as the query against four PRRSV-1 subtype 1 reference sequences (Lelystad virus, BJEU06-1, XJ/PRSSV1/2025, and SCPJ2023), with a 200-nt window, a 20-nt step, and the Kimura 2-parameter model. SHEU-2022 maintained uniformly high similarity (approximately 75–97%) to all four PRRSV-1 references across the entire genome, with no crossover between reference sequences, indicating the absence of recombination. The lower similarity in the nsp2 region reflects the known hypervariability of this region.

**Figure 12:**
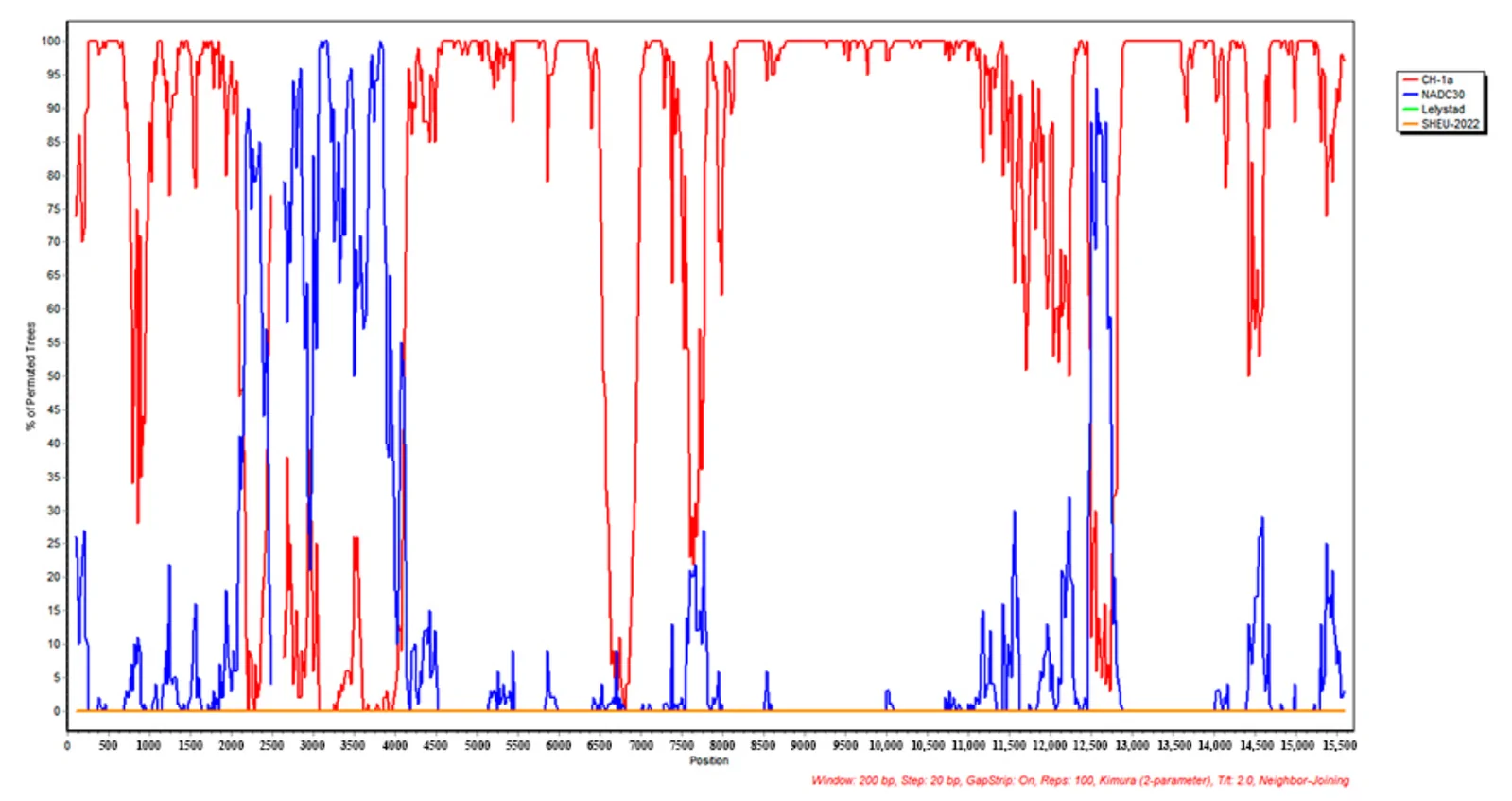
BootScan analysis for inter-genotype recombination between SHCH-2023 and PRRSV-1. The SHCH-2023 sequence was used as the query against CH-1a and NADC30 (PRRSV-2) and Lelystad virus and SHEU-2022 (PRRSV-1), with a 200-nt window, a 20-nt step, the Kimura 2-parameter model, and 100 bootstrap replicates under the neighbor-joining criterion. The two PRRSV-1 sequences showed 0% clustering with SHCH-2023 across the entire genome, confirming that no PRRSV-1/PRRSV-2 recombination occurred between SHCH-2023 and SHEU-2022.

Based on the above phylogenetic analysis results, SHCH-2023 was subsequently identified as a recombinant strain. Although both isolates were from the same clinical case with co-infection, no PRRSV-1/PRRSV-2 recombination between them was detected. In this study, the SHCH-2023 and SHEU-2022 isolates were aligned with 15 reference genomes representing the major circulating lineages of PRRSV-1 and PRRSV-2: for PRRSV-2, CH-1a (AY032626), JXA1 (EF112445), NADC30 (JN654459), IA/2014/NADC34 (MF326985), GXNN20210506 (OK486524), SDLY-27 (OP805381), GX-3 (OR582383), CHah2022 (PP053521), NC2023 (PV342160), and XJ/PRSSV2/2025 (PX789660); for PRRSV-1, BJEU06-1 (GU047344), Lelystad virus (NC_043487), AHB1 (PP068352), SCPJ2023 (PP800763), and XJ/PRSSV1/2025 (PX789659) (Table S1). Sequence alignment was first performed with MEGA 11.0, and then recombination events were analyzed by RDP4 using the seven detection methods (RDP, GENECONV, BootScan, MaxChi, Chimaera, SiScan, and 3Seq). The RDP4 analysis identified two recombination events in the SHCH-2023 genome (Table 4). In both events, the major parent was consistently CH-1a and the minor parent was NADC30: the first region spans SHCH-2023 nt 2131–3573 (corresponding to CH-1a nt 2131–3966, within the Nsp2 region of ORF1a), and the second region spans SHCH-2023 nt 11,833–12,184 (corresponding to CH-1a nt 12,226–12,577, within the GP2/E region). Both events were supported by all seven methods with highly significant P values (*p* < 10^−8^). SimPlot software was used to independently verify this recombination pattern with a 200-nt window, a 20-nt step and the Kimura 2-parameter model (Figure 10), and the results were fully consistent with the RDP4 output, showing a predominantly CH-1a-like genetic background into which two NADC30-derived fragments were inserted, clearly confirming that SHCH-2023 is a natural chimera of the CH-1a lineage carrying two NADC30-derived segments. No intra-genomic recombination event was detected in the SHEU-2022 strain (Figure 11); similarly, no recombination was detected between the SHCH-2023 and SHEU-2022 strains isolated from the same sample (Figure 12).

## 4. Discussion

In this study, two genetically distinct PRRSV strains were isolated from a single sow in Shanghai. Their complete genomes revealed divergent evolutionary trajectories, differential cell tropism, and novel recombination events in the PRRSV-2 isolate.

SHEU-2022 clustered within PRRSV-1 Subtype 1, closest to the domestic isolate 15HEN1-EU (bootstrap 100 in the whole-genome tree; Figure 7). Its 5′UTR and ORF1a/1b sequences shared 78.2–95.9% nucleotide identity with other PRRSV-1 strains (Table 2). ORF6 and ORF2 were the most conserved structural protein genes; ORF3 through ORF5 showed greater variability (Table 3). A 15-nt in-frame deletion was found in the ORF3-ORF4 overlapping region, removing five amino acids from both GP3 and GP4. Together with GP2a, these two glycoproteins form a heterotrimeric complex required for viral infectivity, receptor binding, and membrane fusion [26]. Eight other Chinese PRRSV-1 isolates carry deletions of 3-24 nt at the same site, pointing to a regional evolutionary signature rather than a one-off mutation. SHEU-2022 GP5 has three predicted N-glycosylation sites, one more than LV and Amervac. An extra glycan near the decoy epitope could affect antibody access [27], but this remains a prediction and needs experimental testing. The N protein also carries point mutations in epitopes A and C (Figure 6). Whether these changes alter diagnostic antibody binding is unknown and worth checking.

In contrast to SHEU-2022, the co-isolated PRRSV-2 strain SHCH-2023 fell within lineage 8 (Figure 7). Its ORF5 was 97.6% identical to the classical strain CH-1a, yet its Nsp2 carried the hallmark NADC30-like deletion pattern—131 amino acids lost in three blocks (111+1+19 aa; Figure 8) [28]. This mix of a CH-1a-like genome backbone and ORF5 (lineage 8) with an NADC30-like Nsp2 (lineage 1.8), together with a second, smaller NADC30-derived fragment in the GP2/E region, fits a recombinant history. The 131-aa deletion is a hallmark of NADC30-like strains, which have been associated with prolonged shedding and suboptimal protection by current commercial vaccines [29]. But without challenge experiments, a direct causal connection to pathogenicity cannot be assumed.

The chimeric architecture of SHCH-2023 was further resolved by recombination analysis. RDP4 and SimPlot identified two recombination events, in both of which the CH-1a lineage acted as the major parent (genome backbone) and NADC30 contributed the minor (donor) fragment: one in the Nsp2 region and one in the GP2/E region (Table 4, Figure 10). No PRRSV-1/PRRSV-2 recombination was detected (Figure 12), despite both genotypes sharing the same host. The most likely reason is the low nucleotide identity between PRRSV-1 and PRRSV-2 (~50–60%), which falls well below the threshold for homologous template switching [14]. Incompatibility of the replication complexes, particularly involving the RNA polymerase Nsp9, may pose an additional barrier [8]. However, genotype-specific primers were used for full-genome amplification, so low-frequency inter-genotype recombinants may have gone undetected.

Beyond their genomic differences, the two isolates also showed a clear phenotypic divergence. SHEU-2022 grew only in PAMs, whereas SHCH-2023 grew in both PAMs and Marc-145 cells. This difference is relevant for diagnostics, because Marc-145-based isolation can fail to detect PRRSV-1 [14]. It should be noted, however, that the enrichment strategy may have influenced this result. SHCH-2023 was selectively passaged in Marc-145 cells, and SHEU-2022 was purified in PAMs alone, so the observed tropism difference could partly reflect the isolation method rather than an intrinsic property of either strain. Comparing the original, non-enriched P1 stock in both cell types would help resolve this.

The quantitative data from the original serum sample followed a similar pattern. The farm’s diagnostic qPCR indicated a lower Ct for SHEU-2022 (19.68) than for SHCH-2023 (28.77) in the original serum sample, and the TCID_50_ titer of SHEU-2022 (10^5.47^/100 μL) exceeded that of SHCH-2023 (10^4.37^/100 μL) in PAMs. Ct values reflect viral RNA load in the clinical specimen, whereas TCID_50_ titers represent infectious titer in vitro, and the two metrics are not directly comparable. Although both measures were individually higher for SHEU-2022, the observation comes from a single animal and does not establish a difference in in vivo fitness.

Several limitations should be noted. The sample was small, comprising only six sows from a single farm, which limits how far these findings can be generalized. The cross-sectional design captures a single time point and cannot reveal how viral loads or shedding patterns change over time. Clinical data on vaccination history, parity, the interval between disease onset and sampling, and whether other pathogens were present were not collected. Tissue specimens were unavailable, so the tissue tropism of the two strains in vivo could not be assessed. Controlled challenge experiments in SPF pigs with serial tissue sampling would help address many of these gaps.

To understand how often PRRSV-1 and PRRSV-2 co-infect, and whether they recombine in the field, more farms and regions will need to be sampled. For now, vaccination alone leaves such herds exposed: the modified-live vaccines in use are single-genotype, and no PRRSV-1 vaccine is licensed in China [30]. Multivalent vaccines are hard to develop, yet they will become necessary as herds carry PRRSV-1 and PRRSV-2 together, and increasingly different PRRSV-2 strains as well. Until then, drugs can help fill the gap—macrolides such as tylvalosin and tilmicosin act mainly through immunomodulation [31], and herbal preparations such as Astragalus and Isatis root reportedly inhibit viral replication or activate innate immunity [32,33]. Although PRRSV-2 remains the dominant genotype in China [3], PRRSV-1 deserves equal attention. Together with the recent identification of concurrent PRRSV-1/PRRSV-2 circulation in a single Chinese sow farm [12], and in a Japanese herd [11], our findings underscore the growing coexistence of both species and reinforce the need for continued surveillance and whole-genome sequencing of both genotypes, together with bivalent vaccination strategies [13,34,35] to support disease control and future vaccine design.

## 5. Conclusions

This study reports the co-isolation of PRRSV-1 (SHEU-2022) and PRRSV-2 (SHCH-2023) from a single sow in Shanghai. SHEU-2022 carries a 15-nt deletion in the ORF3-ORF4 overlapping region and an additional predicted N-glycosylation site on GP5; it replicated only in PAMs under the conditions used. SHCH-2023 is a recombinant with a CH-1a-like (lineage 8) genome backbone carrying NADC30-derived fragments in Nsp2 (sublineage 1.8, with the characteristic 131-amino-acid deletion) and in GP2/E; it infected both PAMs and Marc-145 cells, though the enrichment procedure may have contributed to this broader tropism. No PRRSV-1/PRRSV-2 recombination was detected. Although based on a single farm, these findings support the case for herd-level genomic surveillance and the evaluation of bivalent vaccine strategies in China.

## Figures and Tables

**Table 1:** Detailed Information of Primers Used in This Study.

Gene	Primer Name	Sequence (5′→3′)	Position (nt)	Amplicon Size (bp)
*PRRSV-1 ORF7*	ORF7-F	CAACCGAGCATACGCTGTGAG		576
*PRRSV-1 ORF7*	ORF7-R	CTCAAAGGCCACACGCCAA		
*PRRSV-1*	A1-F	TGATGTGTAGGGTATTCCCC	1–1717	1716
*PRRSV-1*	A1-R	CAGTGAACTCCGTTAAGTTTTA		
*PRRSV-1*	A2-F	CRGACGGRTCTTGTGGTTGG	1495–2899	1405
*PRRSV-1*	A2-R	ACAGAGCTGGATTCAGARAG		
*PRRSV-1*	A3-F	ACAGYAGYTCRCCTTTGGAT	2692–4399	1708
*PRRSV-1*	A3-R	ACCTCWGGTGTAGGCTGRTC		
*PRRSV-1*	A4-F	CCATCCATCAAYCACACCAA	4234–5584	1351
*PRRSV-1*	A4-R	GATGTTTGCCAGTAGGCACG		
*PRRSV-1*	A5-F	ACTGCTGCYCATGTGTTGAA	5406–6788	1383
*PRRSV-1*	A5-R	TTTCCTTTCAGYCCCCACATT		
*PRRSV-1*	A6-F	CAGTACATTGAAGCRGCGTAT	6582–8043	1462
*PRRSV-1*	A6-R	GGCACAGTRGGGACATARAG		
*PRRSV-1*	A7-F	TRTCCAAGCAAATAATYCAAGC	7761–9310	1550
*PRRSV-1*	A7-R	YTTCTCCCACATGGACATRA		
*PRRSV-1*	A8-F	GCTCTTGCRTATCACATGAAR	9113–10,602	1490
*PRRSV-1*	A8-R	ACTCGAAATCGAGATGGRCC		
*PRRSV-1*	A9-F	TAAATAAATCCCGRGCACTTGT	10,383–11,813	1431
*PRRSV-1*	A9-R	ACARTGACCCCATTGCATCA		
*PRRSV-1*	A10-F	TTGAAAACACTGAGGATTGGG	11,583–13,287	1705
*PRRSV-1*	A10-R	GCTCATTTCYGAGGCGTAGA		
*PRRSV-1*	A11-F	CTGCCGGTTTCWTGGTCCTY	13,067–14,387	1321
*PRRSV-1*	A11-R	ACAATCTGCATCTGGAAGTGA		
*PRRSV-1*	A12-F	CAAACATGTCGTCCTCGAAG	14,027–15,096	1070
*PRRSV-1*	A12-F	TTTCGGTCACATGGTTCYWG		
*PRRSV-2*	B1-F	TGACGTATAGGTGTTGGCTC	2–1014	1013
*PRRSV-2*	B1-R	AACAAGCKCCACCAGCAGTT		
*PRRSV-2*	B2-F	TTGAAACTGTCCCCGRRGAG	822–2204	1383
*PRRSV-2*	B2-R	YCCAATCAAAGGAGGTGTCC		
*PRRSV-2*	B3-F	GAAAATYRTCAGCCTTTGTCAA	2014–3710	1697
*PRRSV-2*	B3-R	GGGRKTCTYTRGCAGGTTGG		
*PRRSV-2*	B4-F	CTCCCRAAGATGATWCTCGA	3476–5056	1581
*PRRSV-2*	B4-R	AAACGGGTTRGTGCACCACG		
*PRRSV-2*	B5-F	CCACCGGAGTGAAAGTTGAY	4761–6284	1524
*PRRSV-2*	B5-R	TCATTCTCCACAGGAGGAAAA		
*PRRSV-2*	B6-F	AGGCCAGTTTTGTAATGTGRC	6061–7608	1548
*PRRSV-2*	B6-R	ACGTTCATCATACCRAGGGC		
*PRRSV-2*	B7-F	ACAGATGMKTGGGAGTGCCT	7391–8787	1397
*PRRSV-2*	B7-R	TTTCATGAARCCCTGGGTGA		
*PRRSV-2*	B8-F	CCAACCAAGGACATTCAGAG	8584–11,021	1438
*PRRSV-2*	B8-R	CCATGTTGATCTCTTTACAAGT		
*PRRSV-2*	B9-F	ACAAGTYCCGTATAAGCCYC	9825–11,198	1374
*PRRSV-2*	B9-R	TAGTATGACACAACCCCAGG		
*PRRSV-2*	B10-F	CACAGTTTGCTAAACTCCCG	11,006–12,935	1930
*PRRSV-2*	B10-R	TCATGCCCTATCCTRCACCA		
*PRRSV-2*	B11-F	TCCTCCATATTTTCCTCYGTT	12,716–14,198	1483
*PRRSV-2*	R11-R	GAGTAGCGCCAGGACATGCA		
*PRRSV-2*	F12-F	ATTTKACTGGGCAGTGGAGA	13,965–15,412	1448
*PRRSV-2*	R12-R	AATTTCGGCCGCATGGTTCT		
*PRRSV-2 NSP2*	Nsp2-F	ATGTTGTGCTTCCTGGGGTTG		Depends on Subtypes
*PRRSV-2 NSP2*	Nsp2-R	GCTGAGTATTTTGGGCGTGTGAT		

**Table 2:** Length comparison of PRRSV SHEU-2022 strain and other strains.

Name	ID	5′UTR	*ORF1a*	*ORF1b*	*ORF2*	*ORF3*	*ORF4*	*ORF5*	*ORF6*	*ORF7*
SHEU-2022	PQ306310.1	221	7155	4392	750	783	537	606	522	387
15HEN1-EU	KX967492.1	221	7179	4392	750	795	549	606	522	387
BJEU06-1	GU047344.1	221	7176	4392	750	774	528	606	522	387
EUGDHD2018	MK639926.1	222	7160	4357	750	-	549	606	522	387
FJEU13	KP860912.1	221	7176	4392	750	777	531	606	522	387
HeB47	MN927228.1	221	7206	4392	750	798	552	606	522	387
HKEU16	EU076704.1	221	7191	4392	750	774	528	606	522	387
HLJTZJ155-2001	PP330950.1	220	7176	4392	750	774	528	606	522	387
LNEU12	KM196101.1	221	7176	4392	750	795	549	606	522	387
NMEU09-1	GU047345.1	221	7185	4392	750	774	528	606	522	387
NVDC-NM1-2011	JX187609.1	220	7176	4392	750	795	549	606	522	387
TZJ226	OP566682.1	221	7176	4392	750	723	537	606	522	387
Amervac	GU067771.1	221	7191	4392	750	798	552	606	522	387
BE-92V058	MW448197.1	221	7191	4392	750	798	552	606	522	387
Lelystad virus	NC_043487.1	221	7191	4392	750	798	552	606	522	387
SC-2020-1	MW115431.1	210	7185	4389	750	786	540	606	522	387
JXA1	EF112445.1	189	7422	4383	771	765	537	603	525	372
NADC30	JN654459.1	191	7116	4374	771	765	537	603	525	372
SHCH-2023	PQ316100.1	190	7119	4383	771	765	537	603	525	372
VR2332	EF536003.1	-	7509	4374	771	765	537	603	525	372

**Table 3:** Comparison of whole-genome homology between SHEU-2022 strain and other isolated strains.

Name	Number	5′UTR	*ORF1a*	*ORF1b*	*ORF2*	*ORF3*	*ORF4*	*ORF5*	*ORF6*	*ORF7*
15HEN1-EU	KX967492.1	87.3	83.2	83.7	91.2	83.7	85.9	89.1	92.1	86.2
Amervac	GU067771.1	94.5	82.8	83.6	89.6	83.9	83.3	86.1	91.3	84.9
BE-92V058	MW448197.1	94.1	84.4	85.4	91.6	85.5	86.2	84.9	91.1	86.2
BJEU06-1	GU047344.1	94.5	85.6	85.5	92.5	85.8	81.7	89.2	89.6	87.9
EUGDHD2018	MK639926.1	92.7	78.2	79.5	86.1	-	82.5	82	89.4	84.4
FJEU13	KP860912.1	94.1	83.7	84.8	90.8	85.7	85	87.4	91.9	88.4
HeB47	MN927228.1	95	85.1	85.4	92.6	86.5	85.1	88.1	91.9	87.7
HKEU16	EU076704.1	94.1	80.9	82.5	86.9	81.9	80.6	82.5	88.1	86.4
HLJTZJ155-2001	PP330950.1	92.7	82.3	82.7	89.8	85.5	82.5	87.2	89	86.7
LNEU12	KM196101.1	95.9	84.3	84.4	92.1	85.7	86.1	88.9	92.1	88.7
NMEU09-1	GU047345.1	95	78.3	80.3	85.2	83.2	81.7	84.3	89.8	86.4
NVDC-NM1-2011	JX187609.1	94.5	85.2	85.2	91.3	85.3	86.1	88.9	92.1	89.2
TZJ226	OP566682.1	94.1	82.1	83.3	88.1	79.4	84.8	86.1	91.9	86.7
SC-2020-1	MW115431.1	88.2	78.2	80.3	85.3	81.1	80.5	83.4	88.1	85.4
Lelystad virus	NC_043487.1	95	84.5	85.4	91.4	85.9	84.9	84.9	91.5	86.2
VR2332	EF536003.1	-	47.7	58.9	64	61	62.1	59.8	68.3	56.4
JXA1	EF112445.1	46.8	47.9	58.5	63.4	60.6	61.3	59.7	68.5	55.7
SHCH-2023	PQ316100.1	45.4	49.2	58.4	64.5	61.1	63.2	60	69.1	56.4
NADC30	JN654459.1	46.6	48.6	59.4	64.8	62.4	62.2	58	70.4	57.1

**Table 4:** Recombination analysis of SHCH-2023 strain using RDP4 software.

Recombination Event Information of SHCH-2023 PRRSV Isolate (Detected by RDP4 Software).							
Event	Recombinant	Minor Parent	Major Parent	Breakpoints (SHCH-2023, nt)	Breakpoints (CH-1a, nt)	Genome Region	Detection Methods ^a^
1	SHCH-2023	NADC30	CH-1a	2131–3573	2131–3966	Nsp2 (ORF1a)	RDP, GENECONV, BootScan, MaxChi, Chimaera, SiScan, 3Seq
12	SHCH-2023	NADC30	CH-1a	11,833–12,184	12,226–12,577	GP2/E (ORF2)	RDP, GENECONV, BootScan, MaxChi, Chimaera, SiScan, 3Seq

^a^ *p* values—Event 1: RDP 7.7 × 10^−129^, GENECONV 1.2 × 10^−56^, BootScan 6.9 × 10^−108^, MaxChi 4.1 × 10^−44^, Chimaera 5.3 × 10^−41^, SiScan 2.0 × 10^−53^, 3Seq 3.8 × 10^−13^; Event 12: RDP 3.4 × 10^−26^, GENECONV 3.4 × 10^−13^, BootScan 1.7 × 10^−26^, MaxChi 7.4 × 10^−10^, Chimaera 5.5 × 10^−8^, SiScan 3.9 × 10^−12^, 3Seq 3.8 × 10^−13^.

## Data Availability

The genome sequences of SHEU-2022 and SHCH-2023 have been deposited in GenBank under accession numbers PQ306310.1 and PQ316100.1, respectively. Other data generated in this study are available from the corresponding author upon reasonable request.

## Supplementary Materials

The following supporting information can be downloaded at: https://www.mdpi.com/article/10.3390/v18091044/s1, Figure S1. Agarose-gel electrophoresis of the amplified whole-genome products of SHEU-2022 (PRRSV-1); Figure S2. Agarose-gel electrophoresis of the amplified whole-genome products of SHCH-2023 (PRRSV-2); Table S1. Reference strains used in this study.

## Abbreviations

The following abbreviations are used in this manuscript:

BLAST	Basic Local Alignment Search Tool
CPE	Cytopathic effect
DMEM	Dulbecco’s Modified Eagle Medium
GP3/GP4/GP5	Glycoprotein 3/4/5
LV	Lelystad virus
MEGA	Molecular Evolutionary Genetics Analysis
MLV	Modified live vaccine
Nsp	Non-structural protein
nt	nucleotide(s)
ORF	Open reading frame
PAMs	Porcine alveolar macrophages
PBS	Phosphate-buffered saline
PRRS	Porcine reproductive and respiratory syndrome
PRRSV	Porcine reproductive and respiratory syndrome virus
PRRSV-1	PRRSV genotype 1 (European type)
PRRSV-2	PRRSV genotype 2 (North American type)
RDP4	Recombination Detection Program version 4
SPF	Specific pathogen-free
TCID_50_	50% Tissue culture infective dose
UTR	Untranslated region
