# Supplementary material for: Co-Infection of PRRSV-1 and PRRSV-2 in a Single Sow: Complete Genome Characterization Reveals a Novel CH-1a-Backbone Recombinant with an NADC30-Derived Nsp2 in China
Source: Viruses. 2026 Sep 20;18(9):1044. doi: 10.3390/v18091044 (PMC13612029; doi:10.3390/v18091044)
Supplement: Supplementary file 1 [file viruses-18-01044-s001.zip › viruses-4524231-supplementary.pdf]

Supplementary Materials: The following supporting information can be downloaded at: https://www.mdpi.com/article/doi/s1.

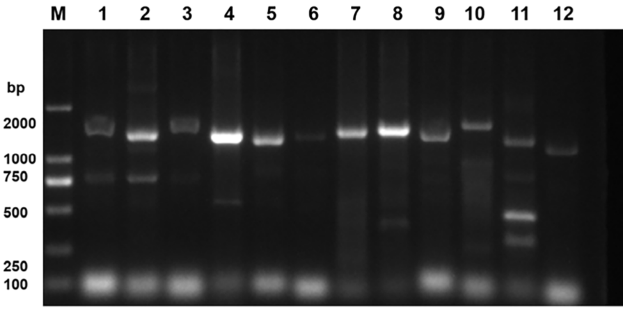

Figure S1. Agarose-gel electrophoresis of the amplified whole-genome products of SHEU-2022 (PRRSV-1). M: DL2000 Marker; A1:1716 bp; A2: 1405 bp; A3: 1708 bp; A4: 1351 bp; A5: 1383 bp; A6: 1462 bp; A7: 1550 bp; A8: 1490 bp; A9: 1431 bp; A10: 1705 bp; A11: 1321 bp; A12: 1070 bp.

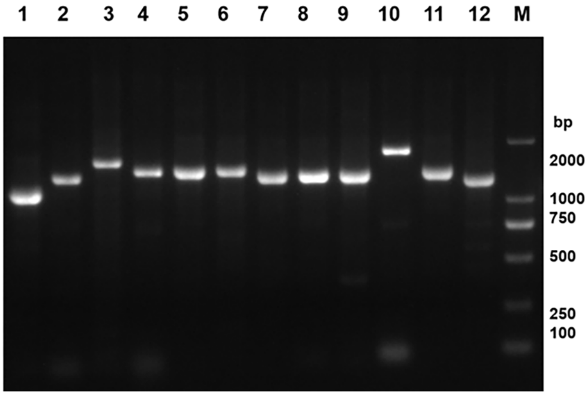

Figure S2. Agarose-gel electrophoresis of the amplified whole-genome products of SHCH-2023 (PRRSV-2). B1: 1013 bp; B2: 1383 bp; B3: 1697 bp; B4: 1581 bp; B5: 1524 bp; B6: 1548 bp; B7: 1397 bp; B8: 1438 bp; B9: 1374 bp; B10: 1930 bp; B11: 1483 bp; B12: 1448 bp; M: DL2000 Marker.

Table S1. Reference strains used in this study.

PRRSV-2 (Betaarterivirus suid 2)		
Strain	GenBank accession	Lineage / sublineage
CH-1a	AY032626	Lineage 8 (classical)
JXA1	EF112445	Lineage 8 (HP-PRRSV)
NADC30	JN654459	Lineage 1 (NADC30-like, 1.8)
IA/2014/NADC34	MF326985	Lineage 1 (NADC34-like, 1.5)
GXNN20210506	OK486524	Lineage 1 (NADC34-like, 1.5)
SDLY-27 [a]	OP805381	Lineage 8 (HP-PRRSV backbone); ORF5 = lineage 1 (NADC34-like, 1.5)
GX-3	OR582383	Lineage 1 (NADC30-like, 1.8)

PRRSV-2 (Betaarterivirus suid 2)		
Strain	GenBank accession	Lineage / sublineage
CHah2022	PP053521	Lineage 1 (NADC30-like, 1.8)
NC2023	PV342160	Lineage 8 (HP-PRRSV)
XJ/PRSSV2/2025	PX789660	Lineage 1 (NADC30-like, 1.8)
PRRSV-1 (Betaarterivirus suid 1)		
Strain	GenBank accession	Subtype
Lelystad virus	NC_043487	Subtype 1
BJEU06-1	GU047344	Subtype 1
AHB1	PP068352	Subtype 1
SCPJ2023	PP800763	Subtype 1
XJ/PRSSV1/2025	PX789659	Subtype 1

a: Recombinant reference strain; backbone lineage 8 (HP-PRRSV), ORF5 derived from lineage 1 (NADC34-like, 1.5).
